# Judgment skills, a missing component in health literacy: development of a tool for asthma patients in the Italian-speaking region of Switzerland

**DOI:** 10.1186/2049-3258-72-12

**Published:** 2014-04-01

**Authors:** Ana Maria Moreno Londoño, Peter J Schulz

**Affiliations:** 1 Institute of Communication and Health, University of Lugano, Via Giuseppe Buffi 13, Lugano CH-6904, Switzerland

**Keywords:** Health literacy, Judgment skills, Asthma self-management, Delphi methodology

## Abstract

**Background:**

Health literacy has been recognized as an important factor influencing health behaviors and health outcomes. However, its definition is still evolving, and the tools available for its measurement are limited in scope. Based on the conceptualization of health literacy within the Health Empowerment Model, the present study developed and validated a tool to assess patient’s health knowledge use, within the context of asthma self-management.

**Methods:**

A review of scientific literature on asthma self-management, and several interviews with pulmonologists and asthma patients were conducted. From these, 19 scenarios with 4 response options each were drafted and assembled in a scenario-based questionnaire. Furthermore, a three round Delphi procedure was carried out, to validate the tool with the participation of 12 specialists in lung diseases.

**Results:**

The face and content validity of the tool were achieved by face-to-face interviews with 2 pulmonologists and 5 patients. Consensus among the specialists on the adequacy of the response options was achieved after the three round Delphi procedure. The final tool has a 0.97 intra-class correlation coefficient (ICC), indicating a strong level of agreement among experts on the ratings of the response options. The ICC for single scenarios, range from 0.92 to 0.99.

**Conclusions:**

The newly developed tool provides a final score representing patient’s health knowledge use, based on the specialist’s consensus. This tool contributes to enriching the measurement of a more advanced health literacy dimension.

## Background

In recent years, increasing attention has been paid to the impact of health literacy on people’s health behavior and health outcomes. The first definitions of health literacy in the early years entailed basic reading, writing, and numeracy abilities needed to perform adequately as a patient [1]. These abilities included being able to read and comprehend medication labels, appointment slips, and other essential health-related materials [2]. The concept has been considerably broadened, and at present, health literacy refers to *the degree to which individuals have the capacity to obtain, process, and understand basic health information and services needed to make appropriate health decisions*[3]. Notwithstanding the wide-spread use of this broader definition, there is a visible discrepancy between its conceptualization and the way it is measured [4]. Nowadays, health literacy skills are still commonly measured by tests for reading and writing abilities. The assessment of only these functional skills is a limitation since those test are unable to capture other dimensions of health literacy that might influence health outcomes as well [5-8].

Among the efforts to broaden the early conceptualization of health literacy, the Health Empowerment Model proposes an additional dimension named judgment skills [9]. These skills are closely related to the conceptualization of *phronesis,* introduced in the context of health literacy by Rubinelly et al., [10]. This focuses on the individuals’ ability to self-examine his own needs, capabilities, and limitations, which in turn would result in preventing him or encouraging him to apply appropriate health decisions [10]. Thus, judgment skills refer to the individual’s abilities to recognize and evaluate when and where to apply a particular knowledge to solve every day problems related with his/her health condition. Such abilities come from the individual’s health knowledge, past experience living with the disease, and individual’s ability to adapt to a changing environment, known as practical intelligence [11]. The inclusion of these judgment skills in the conceptualization of health literacy opens a new window to understand individuals’ health behavior.

Patients regularly have to deal with a variety of situations related to their health condition, including recognition of symptoms, adjustment of medication, and use of treatment devices and therapies. Thus, patients develop their judgment skills over time through an interactive learning process of perception, action, and feedback [11]. Judgment skills are particularly important in the context of self-management of chronic conditions. This is because self-management entails environments characterized by continuous change. These changes include using new therapies entering the market, handling innovative therapy devices, and knowing new guidelines for treatments. Therefore patients need to re-adapt their background knowledge in useful ways to face the new situations.

The self-management of asthma is encouraged in order to monitor symptoms, control allergens that trigger it, and comply with treatment. These practices, when carried out adequately, have improved health outcomes [12].

Asthma is one of the most prevalent chronic conditions worldwide affecting approximately 235 million people around the world [13]. It is an inflammatory disease of the airways and requires a lifelong adherence to medication. Much of the mortality and morbidity of asthma is associated with preventable factors; at least two thirds of asthma deaths and hospital admissions among young people are related to patient denial, lack of recognition of severity, sub-optimal management [14], and low health literacy of patients [15]. Low literacy has been related with high hospitalizations rates, emergency department visits, uncontrolled asthma symptoms, and morbidity [16]. About 6% of the people living in Switzerland suffer from asthma [17]. Half of this population has insufficient asthma control, due to, among others, inappropriate self-management practices [17]. This scenario makes the region an appropriate context to develop and study asthma patients’ judgment skills.

Assessing judgment skills of asthma patients can give an insight into the relationship between information use and self-management practices. Therefore, the aim of the present study is to develop a tool that assesses patients’ judgment skills on asthma self-management competencies.

## Methods

The questionnaire was developed based on the Situational Judgment Test format. This method has been used for years in different contexts such as healthcare, evaluating the clinical judgment of nurses [18], work psychology [19] and personnel selection [20]. The test aims to, among others, assess a person’s ability to apply the appropriate knowledge required for a particular situation [21,22]. The format of the test describes hypothetical situations in which a problem arises, and a list of plausible courses of actions is displayed. The scenarios developed in the present questionnaire describe typical asthma self-management situations where the patient faces a problem, and a list of possible response actions by the patient is provided (Additional file 1: Appendix I). Ethical approval was granted from the ethical committee of the Canton Ticino, Italian-speaking region of Switzerland (i.e. Comitato Etico Cantolane FN132445.Rif.CE2453).

The situational judgment test format was chosen for this study because it allowed the assessment of patients’ abilities on information use rather than assessing knowledge of facts. As stated earlier, one of the biggest limitations of the current health literacy tools is that they only measure functional skills. Several educational programs for asthma patients are aimed primarily at informing patients [16]. However, the connection between knowing facts about a health condition and changes in behavior has not yet been determined [23]. Assessing judgment skills of asthma patients using the situational judgment test format can give an insight into the relationship among knowledge use and the impact of this on self-management practices.

The questionnaire was built in three stages. In stage I, twenty two scenarios with their corresponding response options were drafted. These were built based on scientific literature regarding asthma self-management problems, information from online patients forums, one patients focus group, several patients interviews, and discussions with pulmonologists. In stage II, a Delphi study with a panel of eleven experts on the field of lung diseases was carried out to assess the content and accuracy of the scenarios. In stage III, a scoring scale was generated for the developed questionnaire.

### Stage I: construction of scenarios

A review of scientific literature tackling asthma self-management was carried out. The purpose of this was to identify the main problems encountered by doctors and asthma patients regarding care and treatment of this condition. The databases, ScienceDirect, PubMed, and the Cochrane library were explored using several key words alone or in combination for the search. These key terms were identified by consulting The Global Initiative for Asthma guidelines (GINA) on general competencies and tasks that every asthma patient should have [24]. This included therapy use, symptoms recognition, and compliance. The search was restricted from the early nineties until the present, with two exceptions on the late eighties. These two last studies were included because they also developed a scenarios-based tool on the context of asthma [25,26]. Since some of the situations recreated on those scenarios are common ground on the onset of an asthma attack, information from these former scenarios was added to the description of the developed scenarios in the present study.

Furthermore, different online asthma patient forums were screened for recurrent and communal topics on encountered problems on self-management. Issues that appeared consistently through the literature were grouped into six general topics: *doctor-patient communication*, *medicine usage*, *information seeking*, *triggers avoidance*, *symptoms recognition*, and *exercise*. Under these general topics, several themes were tackled within the single scenarios (Additional file 1: Appendix I).

Twenty-two scenarios were drafted in total. After consulting with a pulmonologist, three scenarios were deleted due to lack of generalization to the majority of the asthma patients, leaving 19 scenarios in the questionnaire.

Following the discussion with the specialist, one patient focus group composed of 4 persons and five patient semi-structured interviews were carried out. Participants were men and women, ranging from 20 to 60 years old, all of them with university level education. Almost 60% of participants were using asthma medicine, 70% had an asthma attack in the previous year, and the majority of them suffered from allergic asthma. All participants live in the Italian-speaking region of Switzerland.

Discussions within the focus group and the interviews were structured around similarities between the scenarios and the participants’ own experiences, descriptions of their self-management strategies, compliance with therapies, and communication with their physicians.

The questionnaire was developed in English and translated into Italian by a native speaker.

#### (a) Doctor-patient communication

The triggers control, the recognition of symptoms, and the appropriate use of medicines are some of the key tasks for asthma control. Doctors play a fundamental role in making patients follow these practices. Several studies reported that the most common causes for non-compliance with therapies is a poor comprehension of the treatment regimen [27], inaccurate recall on how to follow the treatments [28], and mismatches between what physicians say and patients understand [29]. Six scenarios were created on this topic.

#### (b) Medicine usage

Under- and over-use of inhalers is still a big issue in asthma self-management. There are a substantial proportion of asthmatic patients misperceiving the severity of their condition, due to the lack of symptoms recognition and insufficient understanding of what controlled asthma means, resulting in medication misusage [30]. Some patients underestimate symptoms leading them to under-use their medications, while others, over-use their medicines [17,31]. Furthermore, several studies describe that asthma patients tend to reduce their medications when symptoms improve [32], while most patients doubled their inhaled steroid as symptom severity increased [33]. Four scenarios were developed for this topic.

#### (c) Information seeking

This behavior allows patients to be more autonomous and make informed decisions. Several studies show that patients who received asthma information from their physicians actively sought additional asthma information in bookstores, libraries and on-line resources [16]. Patients seeking advice related to health information rely on laypersons and semiprofessional sources [34]. Two scenarios were written for this topic.

#### (d) Trigger avoidance

There are several asthma triggers but not all of them affect individuals in the same way and with the same intensity. Learning to recognize and identify their own susceptibility to triggers is highly recommended by asthma guidelines [24]. Results from the focus group of the present study showed that participants managed their triggers depending on the degree of negative impact on their health status. Thus, if asthma triggers interfered with their lifestyles for instance owning a pet, or smoking, they would have different coping strategies to control triggers. Strategies included increasing the use of medicine, continuing to do what they like unless they felt really sick, or stopping for a while and trying again. Three scenarios were created for this topic.

#### (e) Symptom recognition

A significant proportion of patients underestimate asthma severity, which leads to a higher risk of morbidity or mortality [35]. For asthma patients, it is a priority to be able to recognize symptoms in an early stage. Having the skills to ponder how the health condition is evolving every day and taking the appropriate measures requires experience and knowledge. Two scenarios were developed for this topic.

#### (f) Exercise

This is highly recommended to asthma patients. However, different studies have reported that even patients with mild asthma find exercise challenging, thus, limiting or avoiding these activities, in order to stay away from triggering symptoms [36]. Two scenarios were drafted for this topic.

### Stage II: Delphi study, validation of the scenarios

A Delphi study is an iterative survey conducted to obtain experts opinions and consensus about a topic in their field of expertise [37]. It is carried out individually and anonymously over several rounds. After each round, the results are tabulated and reported back to the expert group. This procedure is repeated until a final agreement on the topic is achieved.

A total of twelve specialists on lung diseases participated in the Delphi study. Participants work in the Italian region of Switzerland, 8 (75%) are specialist in lung diseases and internal medicine, and 4 (25%) in allergy and clinical immunology. In average, the participants have 23 years of work experience as specialists in the field of asthma. Most of them work at the main hospitals of the region and/or have their private practice in the cities of Lugano, Bellinzona, Mendrisio, or Locarno.

The Delphi survey among physicians was used to determine medical opinion on the adequacy of the response options and to validate the scenarios in general. Experts were asked to rate, on a 4-point Likert scale (i.e. adequate, rather adequate, rather inadequate, inadequate), each of the four response options for the 19 scenarios and were encouraged to recommend changes and adjustments in both, response options and scenarios. A response option was considered to reach consensus when at least 60% of votes from doctors lay either on the adequate or the inadequate side of the scale. When a response option achieved consensus, it was shown in the next rounds, but with no possibility to be rated again.

The questionnaire was self-administered, in a paper-pencil format along with an instruction sheet indicating how to rate the response options for each scenario. Table 1 shows an example of one of the scenarios assessed by the Delphi panel.

**Table 1:** Results from the 3 round Delphi study, showing the distribution of doctors’ ratings for each response option of the 19 scenarios, and the round in which final consensus was achieved

**Scenarios’ main topics**	**ICC**	**a**	**b**	**c**	**d**
**1) Exercise**					
**Sc1.** Exercise & rescue medicine	0.93	^§^5/5/-/1	-/1/2/8	-/1/4/6	-/-/4/7
		^¥^**Round 3**	**Round 2**	**Round 3**	**Round 2**
**Sc2.** Exercise & medicine compliance	0.98	-/-/2/9	-/1/6/4	-/-/9/2	9/1/1/-
		**Round 2**	**Round 3**	**Round 2**	**Round 2**
**2) Doctor-patient communication**					
**Sc4.** Doctor’s advice & control medicine	0.98	8/2/1/-	-/-/2/9	9/2/-/-	2/8/1/-
		**Round 2**	**Round 2**	**Round 2**	**Round 2**
**Sc5.** Doctor’s advice & medicine side-effects	0.98	8/3/-/-	1/-/1/9	-/1/9/1	9/2/-/-
		**Round 3**	**Round 2**	**Round 2**	**Round 2**
**Sc12.** Change of medicine recipe	0.92	11/-/-/-	4/5/2/-	2/4/5/-	1/8/2/-
		**Round 2**	**Round 3**	**Round 3**	**Round 3**
**Sc9.** Prescription re-fill	0.99	11/-/-/-	-/-/2/9	-/-/1/10	1/1/7/2
		**Round 3**	**Round 3**	**Round 3**	**Round 3**
**Sc.14.** Asthma symptoms & taking action	0.97	4/7/-/-	9/-/1/-	-/-/7/4	-/-/8/3
		**Round 2**	**Round 1**	**Round 2**	**Round 2**
**Sc11.** Written asthma action plan use	0.99	9/1/-/-	-/-/7/4	-/-/1/10	-/-/1/9
		**Round 1**	**Round 3**	**Round 2**	**Round 1**
**3) Information seeking**					
**Sc6.** Information seeking on-line	0.99	-/-/1/9	6/5/-/-	9/1/-/-	-/1/5/5
		**Round 1**	**Round 2**	**Round 1**	**Round 2**
**Sc7.** Information seeking on medicine side-effects	0.98	10/-/-/-	-/-/6/5	-/3/8/-	2/9/-/-
		**Round 1**	**Round 2**	**Round 3**	**Round 2**
**4) Triggers avoidance**					
**Sc8.** Trigger avoidance & smoking	0.99	10/1/-/-	-/-/-/11	7/4/-/-	-/1/7/3
		**Round 3**	**Round 3**	**Round 3**	**Round 3**
**Sc17.** Trigger avoidance & peak flow meter use	0.99	11/-/-/-	-/-/2/9	-/1/10/-	-/4/6/1
		**Round 2**	**Round 2**	**Round 2**	**Round 3**
**Sc10.** Trigger avoidance & pet owning	0.98	-/-/-/10	10/1/-/-	2/4/5/-	-/-/9/2
		**Round 1**	**Round 2**	**Round 3**	**Round 2**
**5) Medicines use**					
**Sc13.** Control and rescue medicine use	0.99	-/-/1/9	-/1/6/4	-/-/8/3	11/-/-/-
		**Round 1**	**Round 2**	**Round 2**	**Round 2**
**Sc3.** Medicine use & public places	0.98	10/-/-/-	2/8/-/1	-/-/3/8	-/-/1/10
		**Round 1**	**Round 2**	**Round 2**	**Round 2**
**Sc19.** Control medicine use	0.98	9/1/-/-	-/-/6/5	-/-/7/4	-/1/4/6
		**Round 1**	**Round 2**	**Round 2**	**Round 2**
**Sc18.** Asthma symptoms & medicine use	0.98	-/-/5/6	10/-/1/-	-/-/1/9	4/7/-/-
		**Round 2**	**Round 3**	**Round 1**	**Round 2**
**6) Symptoms recognition**					
**Sc15.** Perception of asthma control	0.99	-/-/2/9	-/-/4/7	10/1/-/-	-/-/3/8
		**Round 2**	**Round 2**	**Round 2**	**Round 3**
**Sc16.** Asthma symptoms recognition	0.98	9/1/-/-	-/1/2/7	-/-/2/8	-/-/-/11
		**Round 1**	**Round 2**	**Round 2**	**Round 2**

^§^Distribution of the expert’s rating for each of the response options a, b, c, d. From left to right each number corresponds to a scale-point ranging from Adequate to Inadequate.

^¥^This corresponds to the round in which the response option achieved final agreement.

Content validity was assessed to determine the relevance of the content of the instrument. After the scenarios were drafted, their content was evaluated by two pulmonologists belonging to the Delphi panel. Later on, before starting the second round of the Delphi, each of the panelists was interviewed about the realism of the situations described on the scenarios and the frequency of these problems nowadays. All experts agreed that the content of the scenarios represented most of the common problems encountered in asthma self-management today.

#### First round

**Figure 1:**
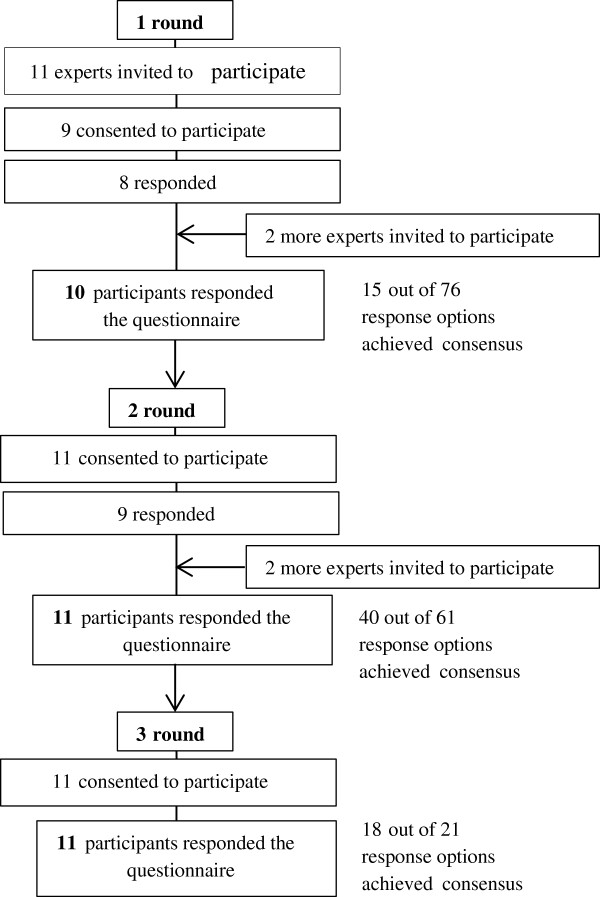
Flow of the recruitment and participation process of the Delphi panelist, & outcomes achieved per round.

For this round, participants were recruited at the annual meeting of pulmonologists working in the Italian-speaking region of Switzerland. Nine out of eleven specialists attending the meeting agreed to participate, and eight of them responded the questionnaire. Therefore, two more specialists from the region were invited to participate, to complete a group of ten experts, as initially planned (Figure 1). These two doctors were contacted through online directories of physicians in Switzerland. Inclusion criteria were having a specialty in lung diseases and working with adult patients in the Italian speaking region. Participation was voluntary and no remuneration was offered.

From the first round, 15 out of 76 response options contained in the questionnaire were rated similarly by more than 60% of the doctors. One scenario depicting the use of a new medicine and its side effects, and another referring to the use of Written Asthma Action Plans (WAAP) resulted in contradictory ratings due to a mismatch between the scenarios and their response options. They were discussed with one of the pulmonologists and replaced. The expert feedbacks and ratings helped to identify drawbacks of some scenarios, including coherence between the scenario and the response options, appropriateness of language used, clarity of the topic, and precision in the description of the symptoms. Amendments regarding these issues were made for the second round questionnaire.

#### Second round

All ten experts who participated in the first round were available for the second round. Since only nine of them answered the questionnaire, two more doctors were invited to participate. Doctors were allowed to sustain their former answers, change them or indicate whether response options were inappropriate for the scenarios.

From this second round, 40 response options out of the remaining 61 achieved expert consensus. Two scenarios tapping the use of WAAP and quitting smoking did not reach sufficient consensus neither in the first nor in the second round; therefore, both were reformulated using information drawn from the focus group and interviews material. The remaining 21 response options that achieved only partial consensus in the second round were discussed with a second pulmonologist and amended for the third round.

#### Third round

Eleven doctors participated in an online survey designed to rate the remaining controversial response options. Only two of these responses did not achieve the established cut off point. The majority of experts who participated in the first and second round responded to this survey.

### Stage III: questionnaire scoring

A ranking of the response options was generated based on the results of the Delphi study. A few months after the Delphi study, doctors were invited to confirm the accuracy of the generated ranking, or to propose a different one in case of disagreement. Nine doctors responded to this survey and only three of the scenarios did not achieve a 100% agreement on the established ranking. Since, two of these scenarios reached a 78% agreement and the other a 67%, no modifications on the ranking were made.

Each response option was scored from 1 (most inadequate) to 4 (most adequate). A sum scale of all 19 scenarios with 4 response options each resulted in a minimum score of 19 and a maximum score of 76. Higher values represent higher judgment skills, indicating improved competency to use health knowledge on asthma self-management (Additional file 2: Appendix II).

## Results

**Figure 2:**
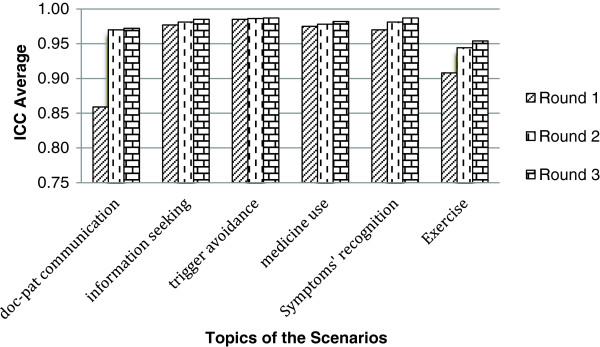
Improvement on doctors’ agreement on the response options rating, through the 3 Delphi rounds.

Intra-class correlation coefficients (ICC) were calculated to measure the similarity of doctors ratings in the three Delphi rounds. The overall ICC for the 76 response options corresponding to the 19 scenarios was 0.97 (Figure 2), and the ICC for the single scenarios ranged from 0.92 to 0.99.

In the final round, only two response options belonging to two different scenarios achieved less than the established cut-off of point of 60% expert agreement. However, they were not modified again, since the ICC coefficients for both scenarios were high, 0.98 and 0.92 respectively, plus the most adequate and most inadequate response options for these scenarios were already identified in the prior rounds. One of the scenarios is about trigger avoidance (pets). The response option stating that the patient will ask the doctor for an alternative solution instead of giving away the pet created divided opinions among the doctors. The other scenario describes a situation of doctor-patient interaction with the doctor changing the patient’s medicine without further explanation. The response option where the patient asks the doctor to prescribe his former medicine instead of the new medicine recommended, created some divided opinions as well.

Table 1 contains the expert ratings per scenario, the round in which the final agreement for the response options were achieved, and the final ICC per scenario.

**Table 2:** Development of tool to assess patient judgment skills on asthma self-management competencies

	**You are fond of animals, and you a have a cat at home. Your doctor has discovered that one of the triggers of your asthma is your cat. Therefore he advised you to give away the cat, because it is damaging your health.**				
	**Please, mark the adequacy level of each of the response options below:**				
		**Adequate**	**Rather adequate**	**Rather inadequate**	**Inadequate**
**a.**	I would not follow the doctor’s advice because I believe that the cat is not related to my asthma.	**□**	**□**	**□**	**□**
**b.**	I would follow the doctor’s advice and give away the cat.	**□**	**□**	**□**	**□**
**c.**	I would ask the doctor if there is any other alternative.	**□**	**□**	**□**	**□**
**d.**	I would increase the use of my medicine to reduce the symptoms caused by my cat.	**□**	**□**	**□**	**□**

Example of a scenario presented to the Delphi participants to rate the adequacy of the response options.

The following is an illustration of how response options achieved consensus in the Delphi study. Scenario: “*You are in a public park talking with your friends, and after some time, you start feeling breathless. Fortunately, you have your rescue medicine with you. What would you do in this situation?”* (a) use the inhaler on the spot, (b) look for a quiet place away from the public for using the inhaler, (c) judge the situation as uncontrollable, or (d) not use the medicine because you consider it is not necessary. Consensus for option (a) was achieved in the first round with a full agreement among the 10 doctors as the most *adequate* response. Consensus for the rest of the options was achieved in the second round. Thus, for option (b), eight in eleven doctors agreed that this was a *rather adequate* answer to the situation. For option (c), ten in eleven doctors agreed that this was *inadequate,* and for option (d), eight experts in eleven concurred that this response was as well *inadequate*. Thus, the level of adequacy of the 4 response options for this scenario was determined (Table 2).

The final questionnaire contains 19 scenarios with multiple response options. Having converging results on the ratings from the experts secures the content validity of the scenarios and response options.

## Discussion

This study describes the development and validation of a tool to measure patient judgment skills in the context of asthma self-management. The questionnaire was developed using the situational judgment test format (SJTs), and it is composed of 19 scenarios with four response options each, addressing the topics of *doctor-patient communication, trigger avoidance, information seeking, medicine use, symptoms recognition, and exercise*. The validation of the tool was conducted in a 3-round Delphi procedure. Twelve experts in the field of lung diseases participated by rating the level of adequacy of the response options. The intra-class correlation coefficient of the questionnaire is 0.97 with coefficients of the single scenarios ranging from 0.92 to 0.99.

Nowadays, patients are requested to have a more participatory role in the healthcare system, helping with the decision-making on treatments, self-managing their health condition, and interacting effectively with healthcare providers, in order to be autonomous patients. This in turn, requires health literate persons capable of carrying out these actions in a competent way. The majority of tools available for assessing health literacy skills tackle reading, writing, and numeracy capabilities [4]. However, there is a common agreement on the need for tools that assess skills beyond the functional ones. The tool developed in this study contributes to fill this gap. The judgment skill tool seeks to assess the patient ability to use health knowledge according to the situation. Assessing these skills, particularly in the context of chronic diseases, is important since self-management plays a key role in the daily care of a health condition. Thus, patients have to embrace constantly changing situations that require skills to use information and knowledge. For instance patients are responsible for judging when to take the medicine, what to do when experiencing symptoms, when to call the doctor or go to the emergency room [25]. Depending on these judgments, the self-management can be directed towards constructive or destructive practices. How this knowledge is applied in different contexts by the patient is something that, to our knowledge, has not yet been assessed. This approach is new in the context of health literacy and might open a new path that contributes to better understanding the impact of health knowledge use on health behavior.

As highlighted before, adequate self-management in asthma has a positive impact on achieving optimal asthma control, improvement of health outcomes, and quality of life [16,38].

The strengths of this study rely on the use of the situational judgment test for the questionnaire, since this has been recognized for successfully predicting individuals’ performance, and appropriate use of knowledge according to the situation [20]. Furthermore, the use of a Delphi procedure to validate the adequacy of the response options from a medical point of view also reinforces the validity of the tool. Although the discussions with asthma patients were also a valuable part of the present work, participants were highly educated and this might have led to overestimating the understanding of the scenarios and reading skills of less educated participants. The SJTs are context-specific instruments, creating the necessity of adapting the existent tool to every particular condition. However, the topics addressed in thescenarios where mainly based on international scientific literature of asthma self-management, thus making it simpler to adapt them to other contexts. Furthermore, the steps taken for the tool development can serve as a guide to develop similar tools for other conditions.

## Conclusions

The developed tool contributes to enriching the measurement of health literacy on the dimension of health knowledge use. Assessing patient’s judgment skills will serve to design better health communication strategies to improve self-management.

## Competing interest

The authors do not have any potential or actual competing interest.

## Supplementary Material

Additional file 1 Appendix I. Click here for file

Additional file 2 **Appendix II.** Scoring sheet for the developed questionnaire. Click here for file
