# Supplementary material for: Judgment skills, a missing component in health literacy: development of a tool for asthma patients in the Italian-speaking region of Switzerland
Source: Arch Public Health. 2014 Apr 1;72(1):12. doi: 10.1186/2049-3258-72-12 (PMC3997838; doi:10.1186/2049-3258-72-12)
Supplement: Additional file 1 — Appendix I. [file 2049-3258-72-12-S1.docx]

| **Topic** | **Description of the 19 Scenarios** |
| --- | --- |
| **Exercise** | **Scenario 1.**The doctor advices the patient to do sports. The patient accepts, and while doing it he starts experiencing some asthma symptoms. What would he do? |
|  | **Scenario 2.** The doctor advices the patient to use the rescue medicine (e.g. Ventolin) before doing exercise. The patient forgets to use it. Thus while doing exercise he start experiencing some asthma symptoms. What would he do? |
| **Medicine usage** | **Scenario 3.** The patient starts to experience some asthma symptoms in a public place. What would he do? |
|  | **Scenario 13**. The doctor advices the patient to use the control medicine (e.g. Seretide) in a daily-basis regime, even during symptom-free periods, and to use the rescue medicine (e.g. Ventolin) only to alleviate symptoms. What would he do? |
|  | **Scenario 18.** The patient has been experiencing strong and frequent asthma symptoms, which did not disappear even after the use of his rescue medicine (e.g. Ventolin). What would he do? |
|  | **Scenario 19.** During the past months the patient has been symptom-free. However, his doctor advised him to continue using his control medicine (e.g. Seretide). What would he do? |
| **Doctor-Patient Communication** | **Scenario 4.** The doctor advises the patient to use a medicine that is cortisone-based. However, the patient feels reluctant use it, since he had experienced in the past several side effects from using this component. Some friends recommend him to use instead alternative medicine. What would he do? |
|  | **Scenario 5**. The doctor advises the patient to use a medicine that is salbutamol-based (e.g. Ventolin). However, the patient feels reluctant to do this, because he knows some people who had experienced bad side-effects from the use of this medicine. What would he do? |
|  | **Scenario 14.** The patient has followed all the directions given by the doctor, regarding the use of his medicine, and he knows what to do in case of experiencing symptoms. Now, he is experiencing strong and frequent asthma symptoms. What would he do? |
|  | **Scenario 11.** The doctor gives to the patient, some time ago, and Action Plan indicating how to use medicines, and what to do in case of symptoms, or an emergency. Now the patient is experiencing strong and frequent asthma symptoms. Therefore, he tries to use his action plan. However, he realizes that he does not remember how to use it. What would he do? |
|  | **Scenario 12.** The doctor give to the patient and action plan, concerning how to use medicines, and what to do in case of symptoms. However, the patient did not fully understood how to use the action plan. What would he do? |
|  | **Scenario 17.** The doctor advises the patient to fix an appointment every six months to evaluate how he is responding to the therapy, and to renew his medicine prescription. However, eight months have passed and the patient (i.e. he has experienced only some mild symptoms) has not yet fixed the appointment with the doctor, and he is running out of his medicines. What would he do? |
| **Information seeking** | **Scenario 6.** The patient consults an online forum because he has been experiencing some side-effects due to the new prescribed medicine by his doctor, and he want to find out if somebody else have passed through a similar experience. A person from the forum advises him to change the frequency and the dosage of the medicine, which is totally opposite to what the doctor advised him. What would he do? |
|  | **Scenario 7.** The patient enters to an online forum to consult about the new prescribed medicine, and he finds some concerning information about the side-effects of this. What would he do? |
| **Triggers avoidance** | **Scenario 8.** The doctor advises the patient to quit smoking and to avoid any tobacco smoke inside the house, despite knowing that some family members that live with him also smoke. What would he do? |
|  | **Scenario 9.** The doctor advises the patient to use a Peak-Flow-Meter to monitor his breathing patterns. What would he do? |
|  | **Scenario 10**. The doctor finds out that the main asthma trigger for the patient is his cat. Therefore the doctor advises the patient to give-up the cat. What would he do? |
| **Symptoms recognition** | **Scenario 15.** The patient has been experiencing a lot of asthma symptoms lately. Therefore he has been using more often his rescue medicine (e.g. Ventolin). Now the patient is with his doctor, and he is asking the patient about his asthma symptoms. What would he do? |
|  | **Scenario 16.** The patient has returned from his vacation on the mountains, where he started to experience some asthma symptoms. Now he is back at home. However, his symptoms have not improved, in fact, they have gotten worse. What would he do? |

Appendix I
