# Supplementary material for: Judgment skills, a missing component in health literacy: development of a tool for asthma patients in the Italian-speaking region of Switzerland
Source: Arch Public Health. 2014 Apr 1;72(1):12. doi: 10.1186/2049-3258-72-12 (PMC3997838; doi:10.1186/2049-3258-72-12)
Supplement: Additional file 2 — Appendix II. Scoring sheet for the developed questionnaire. [file 2049-3258-72-12-S2.docx]

**Appendix II**

**Scoring sheet for the developed questionnaire**

Each response option has a score from 1 (most inadequate) to 4 (most adequate). The scores for the 19 scenarios should be summed up resulting in a minimum score of 19 and a maximum of 76.

Most Adequate = 4

Rather Adequate = 3

Rather Inadequate = 2

Most Inadequate = 1

Minimum score= 19 (e.g. 1 *Most Inadequate ** 19 *scenarios* = 19), *inadequate judgment skills.*

Maximum score =76 (e.g. 4 *19 *scenarios* = 76), a*ppropriate judgment skills.*
